# Attenuation of Cell Mechanosensitivity in Colon Cancer Cells during *In Vitro* Metastasis

**DOI:** 10.1371/journal.pone.0050443

**Published:** 2012-11-30

**Authors:** Xin Tang, Qi Wen, Theresa B. Kuhlenschmidt, Mark S. Kuhlenschmidt, Paul A. Janmey, Taher A. Saif

**Affiliations:** 1 Department of Mechanical Science and Engineering, College of Engineering, University of Illinois at Urbana-Champaign, Urbana, Illinois, United States of America; 2 Departments of Physiology, Physics, and Bioengineering, Institute for Medicine and Engineering, University of Pennsylvania, Philadelphia, Pennsylvania, United States of America; 3 Department of Pathobiology, College of Veterinary Medicine, University of Illinois at Urbana-Champaign, Urbana, Illinois, United States of America; 4 Micro and Nanotechnology Laboratory (MNTL), University of Illinois at Urbana-Champaign, Urbana, Illinois, United States of America; Duke University, United States of America

## Abstract

Human colon carcinoma (HCT-8) cells show a stable transition from low to high metastatic state when cultured on appropriately soft substrates (21 kPa). Initially epithelial (E) in nature, the HCT-8 cells become rounded (R) after seven days of culture on soft substrate. R cells show a number of metastatic hallmarks [1]. Here, we use gradient stiffness substrates, a bio-MEMS force sensor, and Coulter counter assays to study mechanosensitivity and adhesion of E and R cells. We find that HCT-8 cells lose mechanosensitivity as they undergo E-to-R transition. HCT-8 R cells' stiffness, spread area, proliferation and migration become insensitive to substrate stiffness in contrast to their epithelial counterpart. They are softer, proliferative and migratory on all substrates. R cells show negligible cell-cell homotypic adhesion, as well as non-specific cell-substrate adhesion. Consequently they show the same spread area on all substrates in contrast to E cells. Taken together, these results indicate that R cells acquire autonomy and anchorage independence, and are thus potentially more invasive than E cells. To the best of our knowledge, this is the first report of quantitative data relating changes in cancer cell adhesion and stiffness during the expression of an *in vitro* metastasis-like phenotype.

## Introduction

Most cancer deaths are caused by metastasis and not by the primary parent tumor [2], [3], [4], [5], [6]. During metastasis, malignant cancer cells escape from the tumor by detaching from one another or from other cells and the extracellular matrix (ECM) [2], [3], [6], [7]. The escaped cells actively express proteinases and alter their adhesion ligands to degrade and modify their surrounding ECM [3], [4], [5], [8], [9]. Simultaneously, they up-regulate their motility and resistance to apoptosis for successful vascular spread and invasion of distant healthy organs [6], [7], [10]. Concurrently, these cells lower their stiffness [11], [12], [13], [14], i.e., increase their compliance to flow through small capillaries [4], [15], [16]. A quantitative study of the mechanical properties of cancer cells during the early phases of metastasis; however, is lacking [17], [18], [19], [20], largely because of the challenges in detecting the onset of metastasis *in vivo* and the heterogeneity in biochemical and cellular properties of individual tumor cells [3], [17], [21], [22].

We recently discovered [1] that human colon carcinoma cells (HCT-8) can consistently display an *in vitro* metastasis-like phenotype (MLP) when cultured on soft hydrogel substrates with appropriate mechanical stiffness (polyacrylamide gels with Young's modulus: 21∼47 kPa [1], [23]). HCT-8 cells are epithelial (E) in nature. When cultured on soft substrates, they first form distinct epithelial clusters or islands. After 7 days, the cells dissociate from the islands, and assume a rounded shape (R cells). These R cells are highly proliferative, migratory and they significantly down-regulate E-cadherin expression - typical hallmarks of metastasis [1], [24]. Furthermore, E to R transition is repeatable and irreversible [1], [24]. On hard substrates (3 GPa polystyrene substrates), this E to R transition does not occur.

In this study, we first present a detailed investigation of mechanosensitivity of both pre- and post-metastasis-like HCT-8 cells using a gradient stiffness substrate. The study reveals the loss of mechanosensitivity of HCT-8 R cells in contrast to both the E cells and normal fibroblasts. The stiffness of the R cells, measured by AFM, becomes independent of substrate stiffness. In contrast, the stiffness of E cells is correlated with the substrate stiffness. Coulter counter and Bio-MEMS assays reveal that R cells have low homotypic cell-cell adhesion and negligible non-specific adhesion compared to E cells.

## Results

### 1. Weak adhesion between HCT-8 R cells and substrate

**Figure 1:**
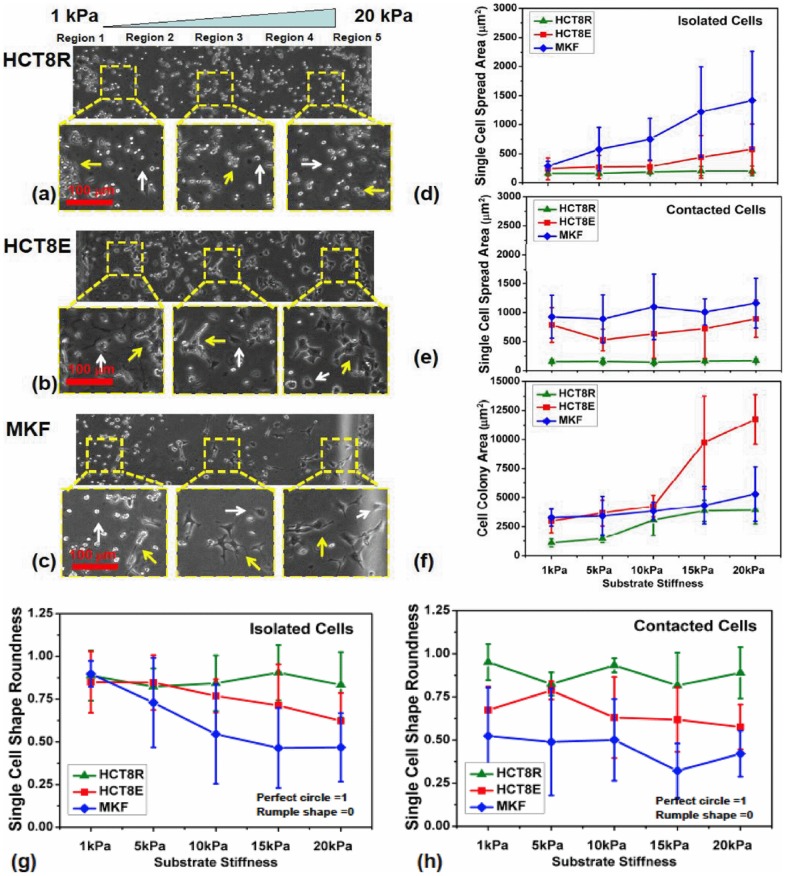
HCT-8 E and R cells and MKF cells cultured on stiffness-gradient PA substrates with stiffness varying continuously from 1 to 20 kPa (left to right). (a–c) Phase contrast images of the harvested HCT-8 R cells, HCT-8 E cells, and normal MKF cells on the gradient-stiffness PA gel substrates. The respective 3 square panels (enclosed by yellow dash boxes) show the representative magnified views on 1–5 kPa, 8–12 kPa, and 15–20 kPa stiffness domains. The white arrows in magnified views indicate the single, non-contact cells, while the yellow arrows indicate the contacting cells in colonies. Scale bars in magnified view panels are 100 μm. (d) The single cells' projected area of 3 cell types across the stiffness range are shown. Here they do not have any contact with their neighboring cells on different stiffness substrates. (e) The spread area of single cells in contact with neighboring cells on different stiffness substrates. (f) The apparent cell colony area of 3 cell types on different stiffness substrates. (g) The cell shape factor of 3 cell types, which are not in contact with their neighboring cells on different stiffness substrates. (h) The cell shape factor of single cells, which are in contact with neighboring cells on different stiffness substrates.

To explore how HCT-8 R cells respond to different physiologically-relevant substrates of varying stiffness, HCT-8 R cells were harvested from soft PA gels, expanded as described in Materials and Methods and then cultured on fresh stiffness-gradient PA gel substrates with stiffness varying continuously from 1 to 20 kPa (Fig. 1a, left to right). The stiffness-gradient substrate is coated with a uniform fibronectin concentration to allow cell attachment to the substrate [25], [26], [27]. For comparison, both HCT-8 E cells and normal Monkey Kidney Fibroblast (MKF) cells, without any prior exposure to PA gels, were plated on the same stiffness gradient substrates and surface functionalization (Fig. 1b and 1c). The normal MKF cells were chosen as control because they are known to be mechanosensitive to substrate stiffness [28]. We found, in contrast to HCT-8 E cells and normal MKF cells, HCT-8 R cells constitutively showed very limited substrate contact areas regardless of substrate stiffness. The R cells' contact area with the substrate is about 40–60% of their apparent projected area. As measured by 3D confocal microscopic imaging, the R cell contact area with substrate is only 49.5±20.9 μm^2^ (n = 34), which is 3.8±0.3 fold smaller than E cells (n = 47), suggesting that R cells have weaker adhesion with the substrate than E cells. The weak adhesion of R cells with substrate is also consistent with the observation that R cells show a smaller projected area, than E cells on the same stiffness substrate (Fig. 1d). The projected area of isolated cells without any neighboring cell contact, of is 1.9 0.6 fold smaller for R cells (n = 68) than E cells (n = 61).

HCT-8 R cells also show a remarkable insensitivity to changing the mechanical-stiffness of their culture substrate. They retain a rounded phenotype and limited adhesion area to substrates regardless of the substrates' stiffness (Fig. 1a, indicated by white arrows; Fig. 1d, 1e and 1g). When the substrate stiffness varied over a 20-fold range, the spread area of single R cells increased only about 27%, (from 156.2±42.1 μm^2^ on a 1 kPa region (n = 62) to 197.9±83.6 μm^2^ (n = 56) on a 20 kPa region) (Fig. 1d). Across the stiffness tested, the increase in R cells' spread area is not as dramatic as that of E and MKF cells. On 5 kPa, 10 kPa and 15 kPa regions, their spread areas are 158.2±40.3 μm^2^ (n = 56), 182.3±32.2 μm^2^ (n = 63), and 190.9±82.5 μm^2^ (n = 57), respectively (Fig. 1d). Also, the R cell shape factor changed only 7% from 0.9±0.2 on a 1 kPa region to 0.8±0.2 on a 20 kPa region (Fig. 1g; The shape factor, S = 4*πA/P^2^, where A is the area of the cell and P is the perimeter. S = 1 for perfect circular shape and 0 for irregular shape), indicating constitutive rounded shape independent of the substrate stiffness. On 5 kPa, 10 kPa and 15 kPa regions, the shape factors of single R cells are 0.8±0.1, 0.8±0.2, and 0.9±0.2, respectively (Fig. 1g). After prolonged culture (60 days), R cells did not show any reversal toward an epithelial morphology on all substrates, regardless of stiffness, even very rigid polystyrene (3 GPa)[1]. In addition, daily recording via video microscopy indicates that R cells show no sign of impairment of proliferative activity even after several months in culture. In contrast, both HCT-8 E cells and MKF cells cultured on the same type of stiffness gradient substrates show obvious sensitivity to the mechanical stiffness of their culture substrate. The individual isolated E cells spread area increases 2.5 fold over 20-fold substrate stiffness change, from 239.6±191.9 μm^2^ on the 1 kPa region to 578.1±429.8 μm^2^ on the 20 kPa region (Fig. 1b, indicated by white arrows). As substrates become rigid, the HCT-8 E cells display a greater spread area, with their spread areas 270.8 201.7 μm^2^ (n = 51), 276.0±104.8 μm^2^ (n = 62), and 442.7±367.7 μm^2^ (n = 55) on 5 kPa, 10 kPa and 15 kPa regions, respectively (Fig. 1b). Their shape factor decreased from 0.9±0.2 on the 1 kPa region to 0.6±0.2 on the 20 kPa region (Fig. 1g). Across other stiffness tested, the single E cells shape factors are 0.8±0.2 (on 5 kPa region), 0.8±0.1 (on 10 kPa region), and 0.7±0.3 (on 15 kPa region), respectively. The mechanosensitivity of MKF is even more pronounced as compared to HCT-8 cancer cells (Fig. 1c). The spread area of individual isolated MKF cells (Fig. 1c; indicated by white arrows) increases 5 fold across the gradient substrate, from 286.4±86.2 μm^2^ (n = 46) on the 1 kPa region to 1421.7±845.7 μm^2^ (n = 31) on the 20 kPa region (Fig. 1d). As the substrate stiffness increases, their spread area increases dramatically, and are 578.1±373.1 μm^2^ (n = 62), 749.9±355.5 μm^2^ (n = 63), and 1218.6±773.5 μm^2^ (n = 59) on 5 kPa, 10 kPa and 15 kPa regions, respectively. Concurrently with increasing substrate stiffness, single MKF cells spread to a more irregular morphology, with their shape factor decreasing from 0.9±0.1 on the 1 kPa to 0.5±0.2 on the 20 kPa regions, respectively (Fig. 1g). On the intermediate stiffness regions, i.e. 5 kPa, 10 kPa and 15 kPa regions, the shape factors of single MKF cells are 0.7±0.3, 0.6±0.3 and 0.5±0.3, respectively. The weak adhesion between HCT-8 R cells and the substrate, as well as the independence of R cell morphology from substrate stiffness, strongly suggest that R cells lose anchorage-dependence and communication with their mechanical microenvironment. This anchorage-independence can potentially promote R cells survival in suspension, which is an essential hallmark of *in vivo* metastasis of cancer cells [2], [3], [4], [16], [22].

### 2. HCT-8 R cells show weak cell-cell adhesion

On stiffness-gradient substrates, both HCT-8 E cells and MKF cells show cell colony formation, especially on stiffer regions (indicated by yellow arrows in Fig. 1b and 1c). The colony size is positively correlated with the substrate stiffness. On substrate stiffness 1 kPa, 5 kPa, 10 kPa, 15 kPa and 20 kPa gels the cell colony sizes of HCT-8 E cells are 2962.2±1000.5 μm^2^, 3662.1±1105.3 μm^2^, 4249.5±919.5 μm^2^, 9736.5±4032.7 μm^2^ and 11748.7±2144.9 μm^2^, respectively (Fig. 1f). For HCT-8 R cells on the same stiffness substrates, the colony sizes are markedly smaller than their E counterparts even when R cells are in contact with neighboring cells for 3 days (Fig. 1a). On substrate stiffnesses of 1 kPa, 5 kPa, 10 kPa, 15 kPa and 20 kPa, the R cell colony sizes are, 1087.4±338.3 μm^2^, 1449.8±343.4 μm^2^, 3062.2±1326.9 μm^2^, 3849.6±919.1 μm^2^ and 3912.1±1183.8 μm^2^, respectively (Fig. 1f). We also observed that inside R cell colonies, the cell-cell contact area is not as extensive as in E cell colonies. R cells appear to be just touching each other at point-contacts (Fig. 1a). These results suggest R cell-cell adhesion is not sufficient for them to form cohesive colonies or cell islands as do E and MKF cells.

Furthermore, it is interesting to note that as HCT-8 E cells or MKF cells undergo homotypic cell-cell adhesion, their individual cell areas and cell shape factor become remarkably less substrate stiffness-dependent (Fig. 1b and 1c, indicated by yellow arrows). Individual cell areas and shape factors of single HCT-8 E cells inside cell islands on 1 kPa gels are 785.6±299.4 μm^2^ and 0.7±0.1, respectively, which is similar to those on 20 kPa gels, 892.8±322.1 μm^2^ and 0.6±0.1 (Fig. 1e and 1 h). Same characteristics are observed on intermediate stiffness, the cell area and shape factor of individual HCT-8 E inside islands are 526.7±187.0 μm^2^ and 0.8±0.1 on 5 kPa gels, 633.9±421.4 μm^2^ and 0.6±0.2 on 10 kPa gels, and 723.1±515.2 μm^2^ and 0.6±0.2 on 15 kPa gels. For individual MKF cells inside islands, their cell area and shape factor are 928.5±374.0 μm^2^ and 0.5±0.3 on 1 kPa gels, 892.8±415.7 μm^2^ and 0.5±0.3 on 5 kPa gels, 1098.1±564.6 μm^2^ and 0.5±0.2 on 10 kPa gels, 1008.8±223.7 μm^2^ and 0.3±0.2 on 15 kPa gels, and 1160.6±429.7 μm^2^ and 0.4±0.1 on 20 kPa gels (Fig. 1e and 1 h). Once these cells establish cell-cell contacts, the E and MKF cells show cell spreading on very soft 1 kPa gels, suggesting the cell-cell signals overwhelm the cell-substrate signals (the left region in Fig. 1b and 1c, indicated by yellow arrows). The majority of HCT-8 R cells; however, remain rounded, with same apparent cell area and shape factor as those of isolated R cells, even when in contact with neighboring cells (Fig. 1a, indicated by yellow arrows). This R cell phenotype results in generally smaller R cell colony area compared to E cell islands consisting of similar cell numbers (Fig. 1f). The individual cell areas and shape factors of single R cells inside R cell colonies on 1 kPa gels are 151.8±33.4 μm^2^ and 1.0±0.1, respectively, and is similar to those on 20 kPa gels (169.6±30.5 μm^2^ and 0.9±0.2), respectively, as well as those of single R cells displaying no cell-cell contacts (Fig. 1e and 1 h). On 5 kPa, 10 kPa and 15 kPa gels, the cell area and shape factor of individual HCT-8 R cells inside islands are 156.2±52.3 μm^2^ and 0.8±0.1, 142.8±47.2 μm^2^ and 0.9±0.0, and 160.7±33.4 μm^2^ and 0.8±0.2, respectively. This unique phenotype persists even after R cells are cultured on the very stiff polystyrene substrates (3 GPa) for prolonged culture times (months); again suggesting weak cell-cell adhesion among R cells. Taken together, these results suggest that during or after E-to-R transition, R cells acquire cell autonomy that is characterized by markedly reduced cell-cell and cell-substrate adhesive contacts.

### 3. R cells have reduced homotypic cell-cell adhesive activity

**Figure 2:**
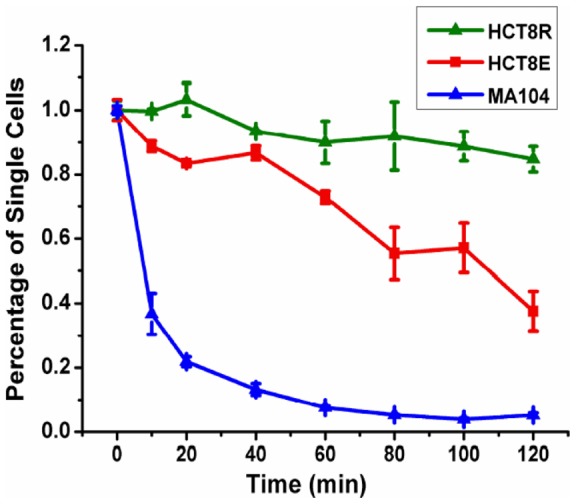
The coulter counter assay is used to measure specific homotypic cell-cell adhesion rates for HCT-8 E and R cells. (a) Comparison of cell-cell adhesion rates of original HCT-8 E cells (never exposed to 21 kPa PA gels), disassociated HCT-8 R cells harvested from 21 kPa PA gels, and normal non-cancerous epithelial Ma104 cells. HCT8 R cells have the lowest cell-cell adhesion. Each data point consists of 3 duplicates, and each duplicate consists of 5×10^5^ cells of respective cell types.

Besides estimating the cell-cell adhesion qualitatively based on their contact morphologies, we further used the coulter counter assay to quantitatively study the functional loss of HCT-8 cell-cell adhesion following E-to-R transition. The coulter counter measures the rate and degree of cell adhesion by quantifying the reduction in the number of single cells in suspension as cell aggregates form as a function of time [1], [29], [30]. The kinetics of specific homotypic cell-cell adhesion for cancerous epithelial HCT-8 E and R cells were measured and compared. Normal (non cancerous) Ma104 epithelial cells were used as a control. We found that disassociated HCT-8 R cells (harvested from 21 kPa PA substrates) displayed a markedly lower rate and extent of cell-cell adhesion as compared to the original HCT-8 E cells cultured on hard polystyrene substrates (Fig. 2). Previous studies have shown that after 120 minutes of incubation, 84.8±4.0% of the HCT-8 R cells remained as single cells, in contrast to 37.6±6.1% of original HCT-8 E cells and 5.2±0.7% of normal Ma104 cells [1]. This remarkable result strongly indicates that the cell-cell adhesive activity of HCT-8 R cells is almost completely lost after they disassociate from E cell islands. This result is consistent with our finding of reduced E-Cadherin expression on R cells [1], [24]. The reduction in cell surface adhesiveness was also seen when non-specific adhesion forces between HCT-8 surfaces and SiO_2_-coated Bio-MEMS probes were measured.

### 4. Cell stiffness changes reflect the mechanosensitivity

**Figure 3:**
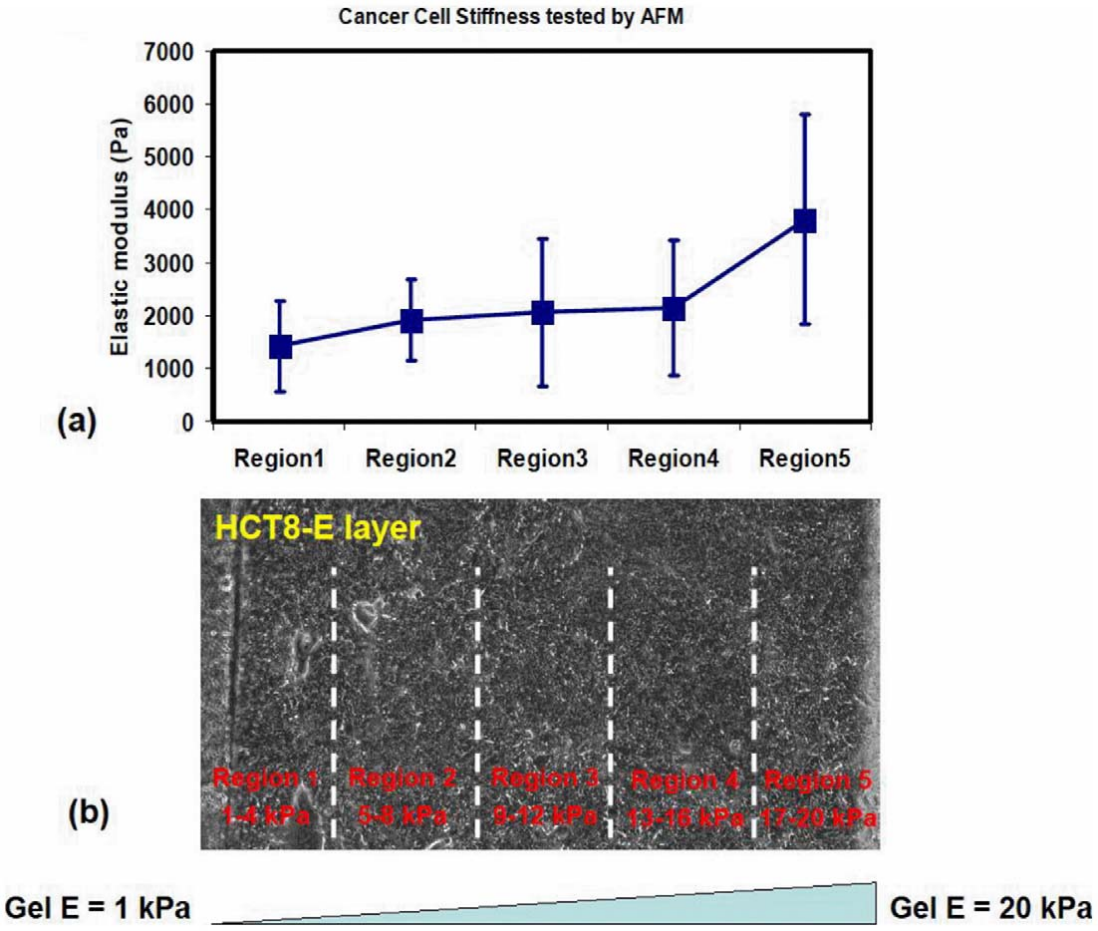
Stiffness and morphology of HCT-8 E cells correlate with substrate rigidity. Using Atomic Force Microscopy, the stiffness of HCT-8 E cells cultured on the gradient substrate is determined. The HCT-8 E cells increase their cell stiffness as the substrates become more rigid. To facilitate the comparison between different cells on same substrate stiffness, five equal-spaced regions across the entire stiffness range are designated: region 1 covers 1–4 kPa, region 2 covers 5–8 kPa, region 3 covers 9–12 kPa, region 4 covers 13–16 kPa, and region 5 covers 17–20 kPa. (a) From region 1 to region 5, the E cell stiffness progressively increases with values 1.42±0.85 kPa to 1.90±0.77 kPa, 2.06±1.39 kPa, 2.15±1.28 kPa, and 3.82±1.98 kPa, respectively. In contrast, on gel substrates with same stiffness gradient, the post-metastatic R cells show almost invariant cell stiffness. (b) Phase-contrast pictures of HCT-8 E cells on gradient PA substrates.

In addition to substrate stiffness-dependent cell morphology changes, HCT-8 E cells also showed varied cell stiffness dependent on culture substrate rigidity. Using atomic force microscopy (AFM), the cell stiffness of HCT-8 E cells cultured on stiffness-gradient substrates is determined by indentation using silicon-nitride cantilevers with a spring constant of 148.14 pN/nm (with consistent cell indentation speed 0.1 μm/sec). Hertz theory (see Materials and Methods) was used to extract the elastic modulus of the indented cells. To facilitate the comparison between different cells on same substrate stiffness, we designated 5 equal-space regions across the entire stiffness range, with region 1 spanning a stiffness of 1-4 kPa, regions 5 with stiffness 5–8 kPa, 9–12 kPa, 13–16 kPa, 17–20 kPa respectively (Fig. 3a). Using AFM, we found HCT-8 E cells increase their cell stiffness as the substrates become more rigid. From region 1 to region 5, HCT-8 E cells stiffness progressively increased from 1.4±0.9 kPa to 1.9±0.8 kPa, to 2.1±1.4 kPa, to 2.2±1.3 kPa, and to 3.8±2.0 kPa, respectively (n = 6∼10 for each region; Fig. 3b). In particular, it is worth noting that the gradient of cell stiffness increase (Fig. 3a) seems to match the gradient of gel substrate stiffness increase. These results are consistent with those previously reported [25], and suggests that HCT-8 E cells are highly responsive to the delicate variation of their microenvironmental mechanical signals. The stiffness of HCT-8 R cells; however, on all the different stiffness substrates, appears invariant at 0.5±0.4 kPa, indicating that R cells have a very limited or no interaction with their mechanical microenvironment. The AFM study also indicated that R cells are mechanically softer than E cells, which potentially may enhance their malleability to allow more efficient invasion of target tissues following *in vivo* metastasis.

### 5. E cell islands show high non-specific adhesion compared to R cells

**Figure 4:**
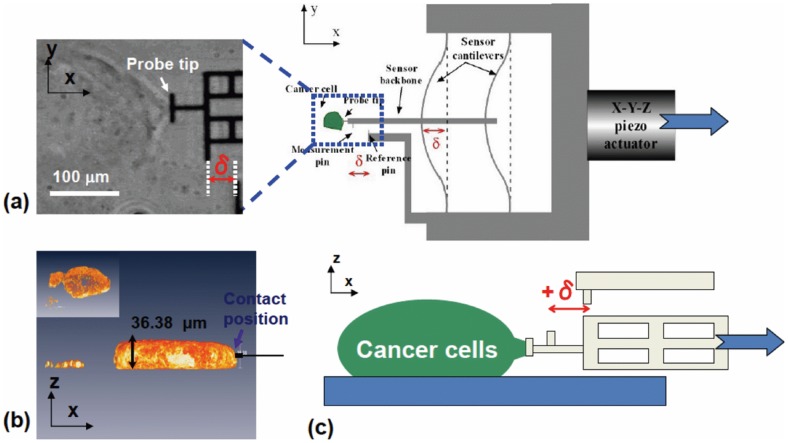
Surface non-specific adhesion of E cell islands measured using a micro-fabricated bio-MEMS force sensor. (a) The non-functionalized micro-fabricated Si force sensor with a flat probe and with known force-deflection relation is manipulated by a high-resolution x-y-z Piezo-stage to contact cell islands' lateral convex surface (on x-y plane). (b) Confocal microscopy of cell islands show the height of islands is on the order of 30∼50 μm. The vertical height of bio-MEMS probe is 5∼10 μm. (c) After a 2-minute contact, force sensor is horizontally pulled away at a constant speed of 2.1±0.4 μm/s. While the cell adhesion between the probe and cell surface hinders retraction of the sensor, the sensor beams deform by δ, giving the force F. Note that the probe is not functionalized. The 2-minute contact between the probe and cells prevents the activation of cell integrins and the formation of any cell focal adhesion, which takes >30 minutes to form.

The surface non-specific adhesions of HCT-8 E cells (4^th^ day of culture on PA gel) and R cells were measured using a micro-fabricated bio-MEMS force sensor (Fig. 4 and Materials and Methods) [1], [30], [31]. The sensor consists of a microcantilever beam with calibrated force-displacement relation (see Materials and Methods). There is a flat probe (width 15 μm and depth 5 μm) attached to the beam, which forms adhesive contact with the cells (Fig. 4a). The sensor is made from single crystal silicon, and is coated with a thin layer of native silicon oxide (SiO_2_). The probe and the sensor are not functionalized. The sensor is manipulated with an x-y-z piezo stage. The flat probe is brought in contact with E-cell islands' lateral convex surface at the boundary. Each E-cell island consists of 100 s of cells with multiple cells stacking at the island periphery (Fig. 4b). After a 2-minute contact, the force sensor is pulled away horizontally from the cell island at a constant speed of 2.1±0.4 μm/s (Fig. 4c). The contact time was chosen as 2 minutes, since prolonged contact duration might result in cellular deposition of ECM on probe and complicate the analysis. Due to the cell-probe adhesion, the sensor beam deforms during retraction, i.e., cells apply a restoring force against detachment. The short contact duration between the cell and the probe prevents the activation of cell integrins and the formation of any focal adhesion on the probe (takes >30 minutes to form [32], [33]). Therefore, only non-specific adhesive interactions can be formed between the cell surface and the SiO_2_-coated probe.

**Figure 5:**
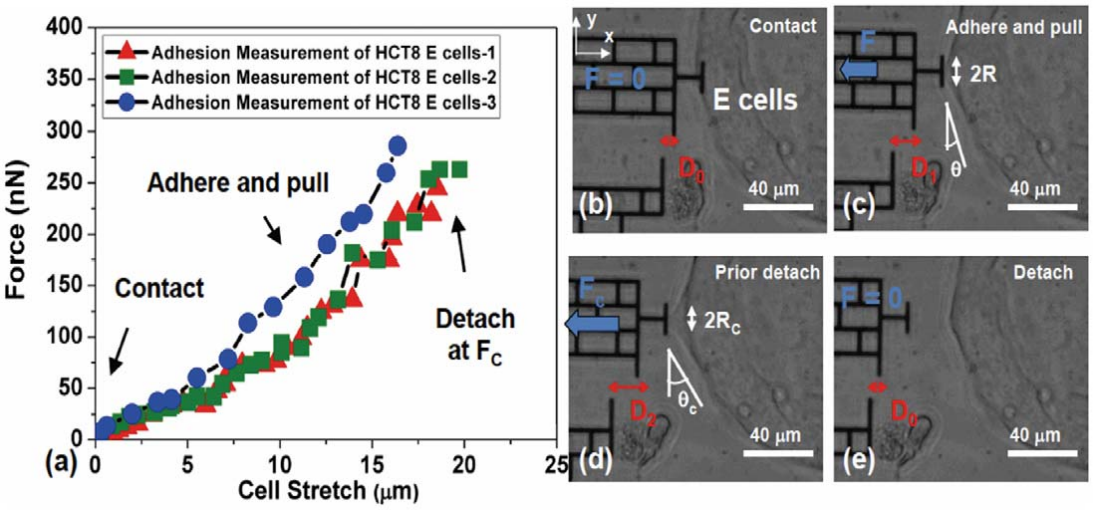
Measurement of E cell island intercellular adhesion by determining detachment force using a Bio-MEMS probe. (a) Intercellular adhesive detachment force of a cell island on the MEMS probe. The force increases monotonically with stretch until detachment. (b–e) Phase contrast images of one typical adhesion experiment. Force is calculated from the deformation of the sensor beam *D* and the force-deformation calibration curve. The critical detachment force, F_c_, is the maximum force on the force-displacement curves. During stretching, the contact angle θ between the probe and the cell island increases, but the contact zone size between the probe and the cell island keeps reducing. Scale bar  = 40 um.

**Figure 6:**
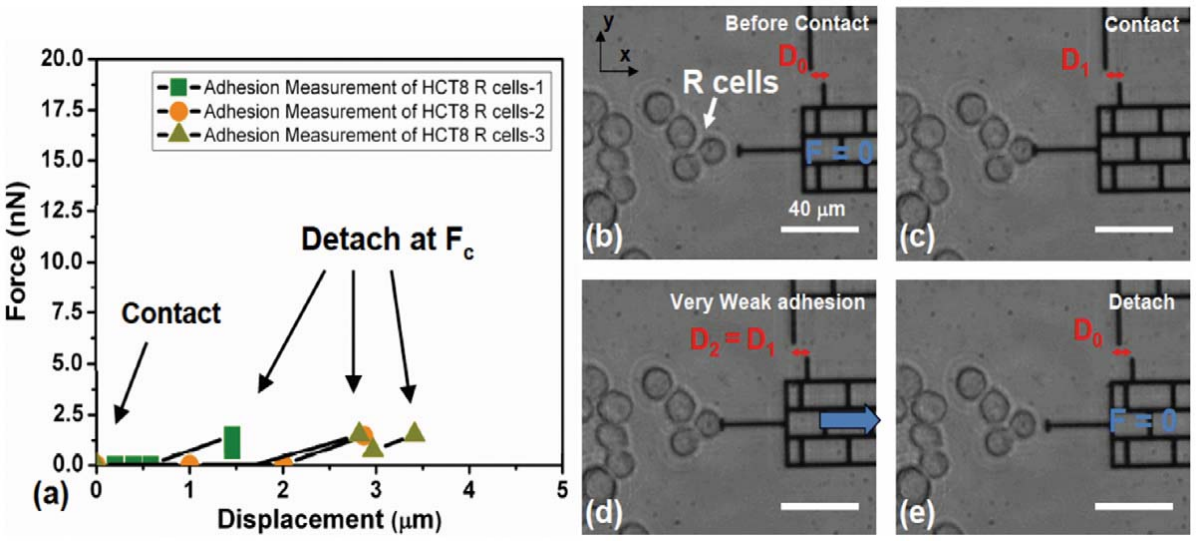
Surface non-specific adhesion of R cells measured by micro-fabricated bio-MEMS force sensor. (a) Adhesive force of R cells on MEMS probe as the probe is moved away from the cells after 2 min contact (n = 25). (b–e). Phase-contrast images of R cells and MEMS probe when non-specific adhesion between them is measured. The maximum detachment force measured is <2.5 nN, while the cell deformation is barely noticeable. Scale bar: 40 μm.

We found that, during retraction of the bio-MEMS sensor, E-cell islands stretch locally by 15–20 μm resulting in a conical shape (see both schematics in Fig. 4c and phase-contrast pictures in Fig. 5). Note this stretch is different from that due solely to membrane tether, which consists of stretching only the phospholipid bilayer. During probe retraction, the cone is continuously stretched with increasing contact angle θ, while the cell contact with the probe drops in a stepwise fashion (Fig. 5b-5d). The increase of force between cell and probe is reflected in the progressive increase of gap between a fixed reference and the probe (from D_0_ to D_1_ and D_2_). Cell force is calculated from the change of gap and force-deformation calibration of the sensor spring. At a critical value of force, F_c_, the cone suddenly detaches from probe (Fig. 5d-e). For E-cells, F_c_ is the maximum force on the force-displacement curves. We consider F_c_ as a measure of cell-probe adhesion. We measured Fc for 12 such cell clusters and obtained F_c_  = 256.3±33.7 nN (n = 12). Similar experiments with R cells show negligible cell-probe adhesion with F_c_  = 1.14±0.13 nN (n = 25; Fig. 6). Hence, R cells have negligible non-specific adhesion compared to E cells and thus appear to be in a “lubricated” state perhaps enabling them to be adapted to passage through vascular capillary beds during *in vivo* metastasis.

## Discussion

To our knowledge, the present study is the first to describe and evaluate the change in mechanosensitivity in human colon cancer cells during a metastasis-like transition produced by solely by changing the mechanical microenvironment during *in vitro* culture. In an earlier paper we reported HCT-8 cells execute an E-to-R transition on 21∼40 kPa stiffness substrates [1]. The present study effectively employs a combinatorial assay system approach using stiffness-gradient substrates, Coulter counter assay, atomic force microcopy (AFM) and Bio-MEMS force sensors to explore the quantitative mechanosensitivity change of human colon carcinoma HCT-8 epithelial E cells as they transit to rounded-shape R cells. We found, triggered by the appropriate substrate rigidity cues, that HCT-8 R cells lose their sensitivity to both the substrate microenvironment as well as their interaction with neighboring R and E cells. As a result, HCT-8 R cells acquire autonomy for survival as anchorage-independent, mobile cells, which is an essential feature of the early events of cancer cell metastasis [3], [4], [5], [6], [16], [20], [30], [34], [35].

The physical properties of culture substrates are found to widely affect the phenotypes and gene expression of a number of normal and cancerous cells [1], [17], [18], [35], [36], [37], [38], [39], [40], [41], [42], [43], [44], [45], [46], [47], [48], [49], [50], [51], [52], [53], [54], [55]. To respond to substrate stimuli, cells adhere to and spread on the substrate followed by sensing and processing both mechanical and chemical signals [26], [37], [44], [46], [49], [53], [55], [56], [57], [58], [59], [60], [61]. As we have previously shown [1], after 7-day culture on soft substrates, HCT-8 cells undergo an E to R transition characterized by R cells dissociating from the parent epithelial cell layer or cell islands. These dissociated R cells show remarkably diminished adhesion (both specific and non-specific [1], [24], [29]) compared to their E cell counterparts. Unlike E cells, the dissociated HCT-8 R cells show substrate-stiffness independent cell-substrate interactions. Their proliferation is not impaired by weak anchorage with the culture substrate or to other cells (Fig. 1). Anchorage independence is a distinguishing feature of metastatic cells [7], [21], [36]. Indeed, our recent *in vitro* basement membrane cell invasion assays indicate that HCT-8 R cells are significantly more invasive than E cells [24].

Our discovery of an E-to-R transition in HCT-8 colon adenocarcimona cells suggests that appropriate substrate mechanical softness may promote or aid in initiation of the early events in cancer cell metastasis, and ironical loss of mechanosensitivity, which could aid in vascular spread to distal tissue target sites. This study reveals that colon cancer cells can attain this trait solely by culture on the appropriately soft substrate. We are currently evaluating whether R cells display enhanced metastatic behavior in animal studies as compared to E cells. If E to R transition correlates with acquisition of enhanced metastatic activity, manipulation of the mechanical microenvironment may serve as an attractive *in vitro* model for investigating the early events of cancer cell metastasis as well as for screening of possible anti-metastatic therapeutic agents.

## Materials and Methods

### 1. Cell culture, microscopy imaging and PA gels preparations

Human colon adenocarcinoma HCT-8 cells (ATCC No.: CCL-244) were cultured in RPMI 1640 (Gibco No.: 23400–062) supplemented with 2 grams of sodium bicarbonate per liter, giving final concentrations of 10% horse serum (Gibco No.: 26050–088), 1× antibiotic-antimycotic (Gibco No.: 15240–062) and 1 mM of sodium pyruvate (Gibco No.: 11360) [1]. Ma104 cells (embryonic African green monkey kidney) were obtained from M.A. Bioproducts and cultured in MEM (Gibco No.: 41500–018) supplemented with 2 grams of HEPES per liter, 2.2 grams of sodium bicarbonate per liter, 1× antibiotic-antimycotic as above, and 5% fetal bovine serum (Gibco No.: 16140). The monkey kidney fibroblast (MKF) cell line (CV-1, ATCC, Manassas, VA) was cultured in a medium with 90% DMEM (ATCC, Manassas, VA), 10% FBS (ATCC, Manassas, VA) and 1× antibiotic-antimycotic (Gibco No.: 15240–062). The cell density before plating was counted with standard hemocytometer. Standard cell culture incubator was used to provide the culture condition with sufficient humidity, 37°C temperature, and 5% CO_2_. An inverted optical microscope (Olympus IX81, Olympus America) with an objective 20× and a high-speed SPOT camera was used to record cell phenotypes and deformation behavior [1], [30], [48], [62], [63], [64]. Polyacrylamide (PA) gels were prepared following the protocols described in the literature [1], [64]. The PA gels of different rigidities were fabricated with varying relative concentrations of acrylamide (Bio-Rad) and N, N'- methylene bis-acrylamide (Bio-Rad) to obtain different cross-link extents. For 21 kPa PA gels, the mol./v concentrations of acrylamide and N, N'- methylene bis-acrylamide are 8% and 0.13%, respectively. All gels were covalently coated with 25 μg/mL fibronectin (BD).

### 2. Bio-MEMS force sensor calibration and experimental setup

We characterized the non-specific adhesion strength of the HCT-8 cells using a novel Bio-MEMS force sensor [30], [63]. Forces were measured using two micromechanical beams with a spring constant 3.48 nN/μm and calibrated using a tungsten microneedle with known stiffness (0.091 N/m) [1], [30], [31], [48], [62], [64], [65], [66], [67]. The tungsten microneedle is 6 mm long and 22 µm in diameter. The force vs. beam deflection characteristics of MEMS force sensor were calibrated using a tungsten microneedle and best fitted to (Eqn. 1):
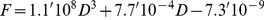
(1)where R-square  = 0.9936. In Eqn. (1), F is the net force acting on the probe along the force sensor backbone and D is the displacement of the probe. Here D  =  D_0_ + δ, where D_0_ is the initial deflection of the sensor beam and δ is the additional deformation due to applied force F. Both F and D are in SI units, Newton and meter, respectively. Before measuring cell adhesion, the sensor was sterilized using Alcohol and DI water multiple times. During the experiment, the T-shaped sensor probe was allowed to contact the cell lateral membrane for 2 minutes and was then moved away horizontally. Due to cell adhesion, the sensor beams deform during retraction by δ, giving the force according to Eqn. (1). Note that the probe is non-functionalized by any extracellular matrix proteins and only has a coating of SiO_2_ on the surface. Therefore, non-specific adhesive interactions were formed between the cell and the SiO_2_-coated probe. The entire pulling process lasts 10–30 seconds.

### 3. AFM calibration of cell island elastic modulus

Atomic force microscopy (Asylum) with silicon-nitride cantilever having a spring constant k = 148.14 pN×nm^−1^ (Veeco) was used to characterize the stiffness of the HCT-8 cell monolayer. A conical tip approximation (Eqn. 2) for the AFM tip was used to extract the substrates' Elastic modulus [1], [48], [68], [69], [70], [71], [72], [73]:
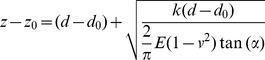
(2)where z and d are the cantilever base PZT displacement and the cantilever tip deflection, respectively. z_0_ is the piezo-controller's vertical position as the AFM tip touches the cell layer's apical surface, and d_0_ is the initial cantilever deflection prior to bending. *v* is the Poisson's ratio for cell layer (*v*  = 0.3∼0.5 in present study). α  = 35° is the half open-angle of cantilever tip. During experiments, the curves of force versus sample indentation were obtained and used to determine Elastic modulus distribution.

### 4. Coulter counter assay

The cells were harvested and individualized by trypsin/EDTA treatment followed by restoration in complete culture medium containing serum to neutralize residual trypsin. Since fibronectin was used for cell adhesion on PA gel substrates and there was no tissue present, trypsin/EDTA (not collagenase) were used to remove cells from culture substrates into single cell state. The cell suspensions were placed in 17×100 mm capped polypropylene tubes (Falcon No.: 352059) and were rotated end over end at 7–8 revolutions per minute in a conventional Labquake shaker (Barnstead/Thermolyne Model No.: 41510) for 1 hour at 37°C to allow recovery of any surface cell adhesion molecules (CAMs) or other proteins. The recovery of CAMs following trypsinization was guaranteed by identifying the increase in cell aggregate number as incubation duration prolongs, as shown in Fig. 2. The pre-incubation time was 1 hour because over-aggregation should be avoided in adhesion-rate assay in order to differentiate the precise adhesion rate kinetic effectively. Portions of the pre-incubated cells (0.3 ml, ∼5×10^5^ cells) were placed in flat bottom vials (Fisher catalog No.: 0333926D) and rotated in a gyratory water bath shaker (G-76, New Brunswick) at 12 rpm at 37°C for 5, 10, 20, 40, 60, 80, 100 and 120 minutes, respectively. At the end of each time period, cells were diluted with 8 mL 0.9% saline and placed on ice to stop further cell aggregation. The number of single cells present at each time point was measured in the Coulter counter as described in [1], [29].

### 5. Immunofluorescent staining and confocal microscopy imaging

Cultures were fixed with 4% paraformaldehyde at 37°C for 30 minutes followed by the 15-minute permeabilization in 0.1% Triton (×100) solution. Rhodamine phalloidin (520/650, red) was used as fluorescent conjugate to stain specifically F-actin filaments. Image-iT™ FX Signal enhancer (Invitrogen, Cat No.: I36933) was used to block all non-specific binding and enhance the imaging quality. The actin structures were imaged using laser-scanning confocal microscopy (Leica SP2, Heidelberg, Germany) with appropriate fluorescent filters, and data were analyzed using Andor IQ software (Andor technology Inc., USA). Multiple images were combined using Amira (Advanced3DVisualization and Volume Modeling) software (Fig. 4b).

### 6. Imaging processing and data analysis

Image stacks processing was performed using ImageJ (NIH) and Photoshop CS3 (Adobe Inc.) software. Statistical data processing and analysis were performed using Office Excel (Microsoft), Origin Pro (OriginLab Corp.) and Matlab (the MathWorks) programs.
